# Effect of Static Magnetic Field on Oxidant/Antioxidant Parameters in Cancerous and Noncancerous Human Gastric Tissues

**DOI:** 10.1155/2016/8608462

**Published:** 2016-05-30

**Authors:** Bahadır Öztürk, Zahide Esra Durak, Süleyman Büber, Ender Hilmi Kocaoğlu

**Affiliations:** ^1^Department of Biochemistry, Selçuk University Medical Faculty, 42131 Konya, Turkey; ^2^The Laboratory of Turkish Public Health Institution, 06100 Ankara, Turkey; ^3^Department of Biochemistry, Ankara University Medical Faculty, 06100 Ankara, Turkey; ^4^Department of Surgical Oncology, Ankara University Medical Faculty, 06100 Ankara, Turkey

## Abstract

*Aim*. To investigate the effects of static magnetic field (SMF) on oxidant and antioxidant parameters of the cancerous and noncancerous human gastric tissues.* Materials and Methods*. Gastric tissues obtained from patients with gastric cancer were used in the study. SMF was created by using two static magnets. Before and after treatment with SMF, oxidant and antioxidant parameters were measured in the tissue samples.* Results*. In the cancerous tissue, superoxide dismutase (SOD) activity was found higher and malondialdehyde (MDA) level was found lower as compared with noncancerous tissue. SMF affects oxidant/antioxidant parameters differently in the cancerous and noncancerous tissues. In this regard, SMF causes increase in SOD activity and decrease in MDA level in the noncancerous tissue. However, it decreases SOD and glutathione peroxidase (GSH-Px) activities and increases MDA level and catalase (CAT) activity in the cancerous tissue. There were no differences between nitric oxide (NO) and nitric oxide synthase (NOS) parameters in or among the cancerous and noncancerous tissues.* Conclusions*. SMF accelerates peroxidation reactions possibly by suppressing SOD and GSH-Px enzymes in the cancerous gastric tissue. This event caused by SMF might play part in the death of cancer cells, which may be a good supportive vehicle for the cancer therapy.

## 1. Introduction

Cancer is one of the leading causes of death in the world. Among them, stomach cancer was the fourth most common cancer and the second highest cause of death from cancer worldwide. It is estimated that, worldwide, it affects about one million people per year [1]. Prognosis is poor because gastric cancer is an aggressive disease and frequently patients present with advanced disease [2]. Its pathogenesis involves diet, obesity, lifestyle, infectious agents, inflammatory process, or genetic variability [3].

Most cancer therapies involve combinations of various therapy protocols. Some negative effects of chemotherapy and radiotherapy have been known obviously. Since classical medical therapies do not result in success for some types of cancers, the scientists have been looking for other ways to treat various types of cancer for some time. Classical therapies can be effective against many types of cancer, but they have toxic potential for normal tissues as well. Because of this fact, high dose of chemotherapy and radiotherapy enough to kill cancer cells completely may be impossible without significant side effects. The use of nonionizing magnetic fields has been shown to have therapeutic potential and warrants further researches on whether it ultimately has a positive role in the therapy of certain forms of human cancer [4].

In recent years, numerous data have been published describing the probable biological interactions of magnetic fields [5–7]. Various biophysical and biochemical effects can be expected to occur when biological systems are subjected to SMFs [8]. Earlier researches reported that effects of SMF on biological systems arise from proinflammatory changes and an elevation in the production of reactive oxygen species (ROS) [9, 10]. Although some molecular mechanisms are supposed for the action of SMF in the cancer process, it seems that there are some other mechanisms unknown in detail yet. As to the subject, we thought that it might be significant to investigate possible effects of SMF on some key enzymes playing part in DNA turnover in cancerous and noncancerous human gastric tissues because, in our previous study, we had established that ADA activity in cancerous and noncancerous gastric tissues was significantly inhibited by the SMF [11].

Therefore, we hypothesized that investigating possible effects of SMF created by permanent magnets on oxidant/antioxidant parameters in cancerous and noncancerous human gastric tissues may make contribution to the attempts of clarifying anticancer potential of SMF.

## 2. Materials and Methods

The study protocol was approved by the Ankara University Ethical Committee of Clinical Research (Decision number 32-690, date: June 13, 2011). Twenty-four cancerous gastric tissues and 24 noncancerous adjacent gastric tissues were obtained from patients with gastric cancer by surgical operation. Tissues were first cleaned by saline solution and then stored at −80°C until the analysis time. In the analysis process, first they were homogenized in saline solution (10%, w/v). After the homogenization, homogenates were centrifuged at 5000 rpm for 30 min to remove debris and to obtain clear supernatant fraction. Then, the analyses were performed in this fraction [12].

In the SMF groups, supernatants were exposed to 100 mT SMF directly for 60 min and then enzyme activity measurements were performed in the samples [13].

Protein concentrations of the tissues were measured by the Lowry method [14]. MDA (nmol/mg) levels, NO (mM) pool (NOO^∙^ + NO_2_^−^), CAT (IU/mg), SOD (U/mg), GSH-Px (IU/mg), and NOS (IU/mg) enzyme activities were measured, respectively [15–19]. MDA levels were measured by the thiobarbituric acid reactive substances method [20]. SOD activity was measured as described before [15]. One unit for SOD activity was expressed as the enzyme protein amount causing 50% inhibition in the nitroblue tetrazolium reduction rate. CAT activity was determined by measuring the absorbance decrease of H_2_O_2_ at 240 nm [16]. GSH-Px activity was measured by following changes in nicotinamide adenine dinucleotide phosphate (NADPH) absorbance at 340 nm [17]. The total NOS activity method is based on the diazotization of sulfanilic acid by NO at acid pH and subsequent coupling to N-(1-naphthyl-ethylenediamine) [18]. Measurement of the NO pool is also based on the same chemical reaction, in which to a greater extent nitric oxide (NO^∙^) and to a lesser extent nitrite anion (NO_2_^−^), but not nitrate anion (NO_3_^−^), give a diazotization reaction with sulfanilic acid. The absorbance of complex one formed with N-(1-napthyl-ethylene diamine) reflects the sum of NO^∙^ and NO_2_^−^ levels in the reaction medium, which is termed the NO pool in the present study. In this method, sodium nitroprusside is used as the chemical standard [19]. In the activity calculations, extinction coefficients of hydrogen peroxide (H_2_O_2_) and NADPH were used for CAT and GSH-Px enzymes, respectively.

### 2.1. Statistical Analysis

Statistical evaluations were made by Wilcoxon test due to nonparametric values and *p* values lower than 0.05 were evaluated as significant.

## 3. Results

As seen from Table 1, SOD activity is higher, but MDA level is lower in the cancerous tissue relative to noncancerous one (*p* < 0.05). There were no differences between other parameters studied in the cancerous and noncancerous tissues.

SMF exerts different effects on the oxidant and antioxidant parameters in the cancerous and noncancerous tissues. SMF significantly increased SOD activity and decreased MDA levels in noncancerous gastric tissues (*p* < 0.05). There were no differences between 2 groups in terms of CAT, GSH-Px, and NOS activities and NO pool in noncancerous group. However, it causes decreases in SOD and GSH-Px activities and increase in CAT activity and MDA levels in the cancerous tissue. There were no significant differences in other parameters between the two groups.

## 4. Discussion

Thus far, there is no clear consensus on the effects of SMFs on human cancers [21]. The available evidence from in vitro and in vivo studies is considered inadequate to decide about the potential impact of SMF exposure because contradictory results were reported regarding the effect of SMF on antioxidant enzymes and oxidative stress. Additionally, the mechanism of action which SMFs exert on cells still remains undisclosed [22].

In biological systems which are exposed to SMF, the process of tissue damage is considered to involve ROS. SMFs cause an increase in the concentration of free radicals and damage of nucleic materials and other macromolecules through the production of oxygen free radicals [23]. The most important ones of the ROS are the superoxide radical (O_2_^∙−^) generated in the mitochondria; H_2_O_2_ produced from superoxide due to the activity of SOD; and peroxynitrite (ONOO^−^) that arose from superoxide and NO. These continuously produced radicals are detoxified by SOD, GSH-Px, and CAT. Endogenous defense mechanisms may lead to failure with overproduction of free radicals and consumption of antioxidants. MDA, an oxidation product of polyunsaturated fatty acids, was accepted as a proven marker of lipid peroxidation [24].

By the virtue of studies performed on SMF impacts to oxidative stress reactions, the probable dangerous effect of SMF is that exposure to SMF might cause oxidative stress by changes in the antioxidant enzyme activity, genetic alterations, and apoptosis by increase in the activity, concentration, and life span of paramagnetic free radicals [25, 26].

Our results show that SOD activity is higher, but MDA level is lower in the cancerous tissue relative to noncancerous one. This might result from the event that oxidant stress created by several factors in the cancerous tissues might lead to compensatory induction in SOD activity, which can prevent peroxidation reactions further.

As to the SMF treatment, our results show that SMF exerts different effects on the oxidant and antioxidant parameters in the cancerous and noncancerous tissues. In this regard, SMF treatment causes increase in the SOD activity and decrease in MDA level in the noncancerous tissue. However, it causes decreases in SOD and GSH-Px activities and increase in MDA level in the cancerous tissue. Diverse specific cellular redox status of different cell types responsible for large variations was observed when they are exposed to magnetic fields [27]. These results suggest that SMF makes opposite effects in the cancerous and noncancerous tissues. It means that SMF decreases increased SOD activity and increases decreased MDA level in the cancerous gastric tissues. However, SMF increases lower SOD activity and decreases higher MDA level in the noncancerous gastric tissues relative to cancerous tissues. We assume that this result may depend on specific properties or compensatory mechanisms regarding biochemical reactions of cancerous and noncancerous tissues.

These different effects created by SMF may supply valuable information to the supportive potentials of SMF in the cancer therapy since it can lead to death of cancerous cells by inducing peroxidation reactions in the cancerous tissues. However, it is obvious that subject needs further long-term experimental and clinical studies to elucidate the effect of SMF before reaching final decision on the subject.

## Figures and Tables

**Table 1:** Median (min–max) values for oxidant/antioxidant parameters in cancerous and noncancerous gastric tissues with SMF and without SMF.

Groups & parameters	Noncancerous gastric tissue (*n* = 24)		*p*	Cancerous gastric tissue (*n* = 24)		*p*
	Without SMF	With SMF		Without SMF	With SMF	
CAT (IU/mg)	5.33 (0.95–13.56)	6.04 (1.95–12.31)	n.s.	4.53 (2.31–9.51)	6.09 (3.17–10.12)	<0.01

MDA (nmol/mg)	0.44 (0.08–4.37)	0.28 (0.09–2.28)	<0.05	0.34^*∗*^ (0.05–1.69)	0.44 (0.05–3.28)	<0.01

SOD (U/mg)	1.39 (0.29–4.64)	1.98 (0.40–4.23)	<0.05	1.59^*∗*^ (0.59–4.25)	1.20 (0.4–2.58)	<0.05

GSH-Px (IU/mg)	0.021 (0.003–0.041)	0.015 (0.003–0.039)	n.s.	0.021 (0.004–0.065)	0.012 (0.008–0.092)	<0.05

NO (mM)	29.38 (19.5–45)	29.25 (12.75–40.75)	n.s.	31.25 (20–41.5)	28.38 (15–47.25)	n.s.

NOS (IU/mg)	1.27 (0.34–2.95)	1.22 (0.31–2.53)	n.s.	1.53 (0.32–3.68)	1.42 (0.03–3.52)	n.s.

^*∗*^Significantly different than noncancerous gastric tissue without SMF (*p* < 0.05).
